# CD19-targeted BiTE expression by an oncolytic vaccinia virus significantly augments therapeutic efficacy against B-cell lymphoma

**DOI:** 10.1038/s41408-022-00634-4

**Published:** 2022-02-28

**Authors:** Wen Lei, Qian Ye, Yuanyuan Hao, Jie Chen, Yu Huang, Liu Yang, Shibing Wang, Wenbin Qian

**Affiliations:** 1 Department of Hematology, Key Laboratory of Cancer Prevention and Intervention, China National Ministry of Education, Key Laboratory of Molecular Biology in Medical Sciences, The Second Affiliated Hospital, Zhejiang University School of Medicine, 310009 Hangzhou, Zhejiang P. R. China; 2 Cancer Institute, The Second Affiliated Hospital, Zhejiang University School of Medicine, 310009 Hangzhou, Zhejiang China; 3 Hangzhou RongGu Biotechnology Limited Company, 310056 Hangzhou, Zhejiang P. R. China; 4 Academy of Chinese Medical Sciences, Zhejiang Chinese Medical University, 310053 Hangzhou, Zhejiang P. R. China; 5 Cancer Center, Department of Medical Oncology, Zhejiang Provincial People’s Hospital, Affiliated People’s Hospital, Hangzhou Medical College, 310014 Hangzhou, Zhejiang P. R. China; 6 Cancer Center, Molecular Diagnosis Laboratory, Key Laboratory of Tumor Molecular Diagnosis and Individualized Medicine of Zhejiang Province, Zhejiang Provincial People’s Hospital, Affiliated People’s Hospital, Hangzhou Medical College, 310014 Hangzhou, Zhejiang P. R. China

**Keywords:** Cancer immunotherapy, Cancer immunotherapy

## Abstract

Immunotherapy with CD19-targeting bispecific T-cell engagers (CD19BiTEs) has demonstrated highly effective killing of cancer cells in patients with precursor acute lymphoblastic leukemia and non-Hodgkin’s lymphomas. However, there are some drawbacks to this therapy, such as toxicity, short half-life in the serum, and immunosuppressive tumor microenvironment that could limit the use of CD19BiTEs in the clinic. Here, we generate an oncolytic vaccinia virus (OVV) encoding a CD19-specific BiTE (OVV-CD19BiTE). We demonstrate that OVV-CD19BiTE’s ability to replicate and induce oncolysis was similar to that of its parental counterpart. Supernatants from OVV-CD19BiTE-infected cells could induce activation and proliferation of human T cells, and the bystander effect of the virus was also demonstrated. In vivo study showed that OVV-CD19BiTE selectively replicated within tumor tissue, and contributed to a more significantly increased percentage of CD3, CD8, and naïve CD8 T subpopulations within tumors in contrast to blinatumomab. More importantly, treatment with OVV-CD19BiTE both in vitro and in vivo resulted in potent antitumor activity in comparison with control OVV or blinatumomab, a first-in-class BiTE, thereby resulting in long-term tumor remissions without relapse. The study provides strong evidence for the therapeutic benefits of CD19-targeting BiTE expression by OVV, and suggests the feasibility of testing the approach in clinical trials.

## Introduction

With the major progress made in recent years, CD19-based immunotherapy has changed the treatment pattern of relapsed or refractory (R/R) B-cell malignancies [1, 2]. Bispecific T-cell engagers (BiTEs) are novel immunotherapeutic molecules that consist of an anti-CD3 single-chain variable fragment (scFv) fused to the scFv targeting antitumor-associated antigen via a flexible linker. BiTE-mediated T-cell-antigen-positive target cell crosslink triggers the formation of cytolytic synapses, which results in tumor-specific cell lysis, and can overcome elements of tumor-associated immunosuppression that limit physiologic immune responses, leading to reactivation and proliferation of exhausted tumor-specific T cells [3, 4]. Blinatumomab, a CD19/CD3 BiTE, has been approved in the R/R B-cell precursor acute lymphocytic leukemia (ALL) based on the Phase III TOWER study that demonstrated improved overall survival (OS) and complete remission (CR) when compared to standard of chemotherapy [5, 6]. It has also shown promise for use in non-Hodgkin lymphoma (NHL) [7]. However, NHL patients with extramedullary involvement may be more resistant to BiTE therapy [8], suggesting a limited capacity of BiTEs to penetrate the tumors. Other limitations are that the short half-life of blinatumomab requires a continuous infusion over 6–8 weeks which is the major hurdles of use in the clinic [6, 7], and the majority of patients receiving this drug had grade 3 or higher adverse events (AEs) [5].

To enhance the efficacy of BiTE agents and decrease unwanted AEs due to systemic administration, local delivery of BiTEs at the tumor site would provide consistent, durable therapeutic responses against cancers without systemic toxicity. Oncolytic viruses (OVs) selectively infect, replicate in, and destroy tumor cells, representing a new class of therapeutic agents. Clinical trials have demonstrated the safety and potential of OVs [9]. Moreover, it has been well known that OVs also induce antitumor immune responses and revert immune suppression, which functions to eradicate cancer cells within the tumor microenvironment (TME) [3, 10, 11]. We and others have previously demonstrated that OVs can be “armed” to express therapeutic transgenes, and secrete them from infected cancer cells, thereby significantly enhancing its antitumor activity [12–15]. Additionally, several novel OVs armed with BiTE, such as EGFR, EphA2, and FAP, have been shown to enhance T-cell activation and proliferation, increase the persistence and accumulation of T cells in tumors, and result in higher antitumor efficacy compared to the parental virus [16–19].

Among OVs, vaccinia virus possesses many positive attributes that make them a more attractive platform for genetically modified OV-based immunotherapy. Vaccinia viruses have a large genome with the capacity for arming multiple therapeutic genes and replicating in the cytoplasm, therefore, minimizing any chance of integration into the host genome [15]. It has a long track record of safety when used as a smallpox vaccine [20]. More importantly, intravenous delivery of oncolytic vaccinia virus (OVV) in cancer patients showed persistent infection in metastatic tumors and induction of anticancer immune responses [21, 22].

In this study, we generated an OVV encoding bispecific T-cell engagers (OVV-CD19BiTE) that bound both to human CD3 and a lymphoma cell surface antigen CD19 and assessed its efficacy in vitro and in vivo. Furthermore, we investigated the possible mechanisms of efficacy with attention to T-cell recruitment, activation, and differentiation.

## Methods

### Cell lines and cell culture

The cell lines used in this study included Raji (Human Burkitt’s lymphoma cell), Farage (Human B-cell lymphoma cell), Pffeifer (Diffuse large B-cell lymphoma cell), Nalm-6 (Human B-cell lymphocyte leukemia cell), Jurkat (Human T-cell lymphocyte leukemia cell), CEM (Human T-cell lymphocyte leukemia cell), HEK293 (human embryonic kidney cell), and Hela-S3 (Human cervix carcinoma cell), K562 (Human chronic myelogenous leukemia cell), and K562/CD19 (K562 cells transfected with human CD19 antigen). HEK293 and Hela-S3 cells were cultured in Dulbecco’s modified Eagle’s medium (DMEM; Cat# 11965092, Gibco-Thermo Fisher Scientific, USA) supplemented with 10% fetal bovine serum (FBS; Cat#16000044, Gibco). Raji, Farage, Pffeifer, Nalm-6, Jurkat, CEM, K562, and K562/CD19 cells were cultured in RPMI 1640 medium (Cat# 11875093, Gibco) supplemented with 10% FBS (Cat#16000044, Gibco). Hela-S3 cells were cultured in suspension in a serum-free medium (Cat# H740KJ, Basalmedia, Shanghai, China) in spinner flasks (Cat# TCB002002, Jetbiofil, Guangzhou, China). All cells were incubated at 37 °C with 5% CO_2_ atmosphere.

### Recombinant OVV construction, purification, and expansion

The shuttle plasmid pRGB001 was synthesized by GenScript (Nanjing, China). In this plasmid, the reporter gene EGFP and the screening gene guanine-hypoxanthine phosphoribosyl transferase (GPT) are linked by a T2A peptide sequence and are under the control of the p-7.5k early/later promoter. There is another synthesized p-se/l promoter back-to-back with the p-7.5k promoter to control the expression of foreign genes. These chimeric genes are flanked by the left (TKL) and right (TKR) fragments of the VV thymidine kinase (TK) gene. CD19-targeted BiTE was constructed by linking a scFv recognizing human CD19 (clone FMC63) to a scFv recognizing CD3ε (clone OKT3) via a flexible glycine-serine linker (G4S). The anti-CD19 and anti-CD3 variable regions were connected by a (G4S)3 and a (G2S)4GG linker, respectively. The CD19BiTE was arranged VHCD19-VLCD19–VHCD3–VLCD3 and included the signal peptide (SP) from the mouse Ig heavy chain and a FLAG tag at the N- and C-terminus of the protein, respectively. Finally, the CD19BiTE construct was optimized for human codon usage and synthesized by Genscript and subclone into the pRGB001 plasmid to construct a recombinant shuttle plasmid (pRGB001-CD19BiTE) for expressing the CD19BiFTE under the control of the p-se/l promoter.

To generate recombinant OVV, a Western Reserve (WR) strain (ATCC VR-1354) was used as a parental virus for homologous recombination. In brief, HEK293 cells were infected with WR at a multiplicity of infection (MOI) of 0.1 for 4 h and then transfected with the corresponding shuttle plasmid using Lipofectamine 3000 transfection reagent (Cat# L3000015, ThermoFisher, USA). The cell extraction solution was used to infect the HEK293 cells in the presence of 25 mg/ml mycophenolic acid (MPA; Cat# A600640, Sangon Biotech, Shanghai, China), 250 mg/ml xanthine (Cat# A601197, Sangon Biotech), and 15 mg/ml hypoxanthine (Cat# A500336, Sangon Biotech). After three cycles of screening, EGFP-positive plaques were isolated, resuspended, and further infected HEK293 cells for two cycles of plaque purification. After completing the first and second rounds of plaque purification, the following primers were used to amplify the target gene and the viral TK gene to identify whether the recombinant virus was adulterated with the parental vaccinia virus. Primer of target gene: 5′-GGCAACCGCAACAGGAGT-3′, 5′- CGGAGTCTCGTCTGTTGTGG-3′; Primer of TK: 5′-TGTGAAGACGATAAATTAATGATC -3′, 5′-GTTTGCCATACGCTCACAG-3′. Recombinant vaccinia virus successfully screened by plaque purification was further expanded by Hela-S3 cells in six-well plates, cell culture dishes, and cell culture spinner flasks

### Replication of the oncolytic vaccinia virus in lymphoma cells

Tumor cells (Raji, Pffeifer, Farage, and K562/CD19) were seeded in a 24-well plate at 6 × 10^4^ cells per well and placed in a 5% CO_2_ incubator at 37 °C, and then infected with recombinant OVVs at MOIs of 0.5. The cells were harvested after 24, 48, 72, and 96 h and then disrupted by three freeze-thaw cycles, and centrifuged at 1000 × *g* for 30 min. The virus supernatants were collected and the virus titer was determined by an TCID50 method.

### CCK8 assay

For the CCK8 assay, Raji, Farage, Pffeifer, and K562/CD19 cells were seeded in a 96-well plate at 8 × 10^3^ cells per well and then infected with OVV at indicated MOIs in triplicate. After 72 h incubation, 20 μl of CCK8 solution (Cat# C0037, Beyotime, Shanghai, China) was added to each well of the plate and incubated for 4 h. The absorbance was measured at 450 nm using a microplate reader (Spectra-Max M3, Molecular Devices, USA). The cell viability is calculated according to the following formula: cell viability (%) = (A treatment − A blank)/(A control − A blank) × 100%.

### Western blot

The protein samples were collected from supernatants of OVV or OVV-CD19BiTE-infected HeLa-S3 cells. BCA assay was then conducted to measure protein levels in these samples based on the provided directions. Equal protein amounts were separated via 10–15% SDS/PAGE and transferred onto PDF membranes that were probed with anti-FLAG (Abcam, ab205606). Blots were then probed with HRP-linked secondary antibodies (HuaAn Biotechnology) for 1 h.

### Binding assays

To detect whether the secreted CD19BiTE could bind to the recombinant CD3ε protein (Cat# CDD-H52W1, Acro) and CD19 protein (Cat# CD9-H52H2, Acro), a FLAG-linked Elisa assay was performed. Briefly, 96-well plates were coated with CD3ε or CD19 protein at a concentration of 10 mg/ml. Then, supernatants of OVV-infected Hela-S3 cells were collected and added to the coated wells and incubated at 4 °C for 12 h. After that, the cell culture supernatant was removed, washed three times with 1 × TBS buffer, BiTE binding CD3ε and CD19 protein was determined by ELISA assays using anti-FLAG M2-HRP antibody (Cat#A8592, Sigma–Aldrich).

Binding assays were also performed with CD19^+^ tumor cells: K562/CD19, Raji, Farage, Pffeifer, Nalm-6 cells or CD3^+^ cells: Jurkat, CEM, Human T cells. Target cells (1 × 10^5^) were incubated on ice for one hour with the OVV-infected supernatants. BiTE binding was determined by flow cytometry using APC anti-DYKDDDDK Tag antibody (Cat# 637308, Biolegend, USA).

### Enzyme-linked immunosorbent assay (ELISA)

Human T cells were co-cultured with or without K562, K562/CD19, or Raji cells, respectively, in the presence of the OVV or OVV-CD19BiTE supernatants (100 μl) or blinatumomab (100 ng/ml) for 24 h. Then supernatants were obtained from this co-culture system. Samples were centrifuged at 600 × *g* for 5 min at room temperature and the supernatants were collected. Human IFN-ɣ, TNF-α, and IL-2 in the supernatants were quantified using the ELISA MAXTM Standard Set (Cat#430115, 430216, 431815, Biolegend, USA) according to the manufacturing protocol.

### Quantitative polymerase chain reaction (qPCR)

Frozen tumor samples were disrupted using a mortar and pestle under liquid nitrogen. RNA and DNA were isolated from approximately 25 mg of homogenized tissue with the DNA/RNA/protein kit (IBI Scientific). RNA samples were treated with the TURBO DNA-free kit (Thermo Fisher Scientific) to remove traces of genomic DNA. RNA (1 μg) was retrotranscribed with the High-Capacity cDNA Reverse Transcription kit (Thermo Fisher Scientific). Real-time analysis was performed in a LightCycler 480 Instrument II (Roche). To quantify the viral genomes and CD19BiTE transcripts in the tumor, 100 ng of DNA and 40 ng of cDNA in the presence of SYBR Green I Master (Roche) were used, respectively. PCR conditions were: 95 °C 10 min, 40 cycles of 95 °C 15 s, 60 °C 1 min and 72 °C 7 min. Viral genome primers were A56F: 5′-: CTGGATCTACACATTCACCGGA-3′ and A56R: 5′-ATGAACCGCAGCGTCAAACG-3′ and CD19BiTE primers were qBiTEF: 5′- GGCAACCGCAACAGGAGT -3′ and qBiTER: 5′- CGTAGTCTGGCAGGCTCA -3′.

### Preparation of peripheral blood mononuclear cells and T-cell isolation

All experiments were approved by the ethics committees of the Second Affiliated Hospital, College of Medicine, Zhejiang University. Every donor signed the informed consent. Blood samples were obtained from the healthy donors. And peripheral blood mononuclear cells (PBMCs) were isolated by Ficoll-density gradient centrifugation and resuspended in AIM-V medium (Gibco, Grand Island, NewYork, USA) with 10% FBS for incubation in a 37 °C, 5% CO_2_ humidified incubator. For preparing the T cells, PBMCs were isolated using T Cell Isolation Kit (MiltenyiBiotec, BergischGladbach, Germany), then resuspended in AIM-V medium containing IL-2 (300 IU/ml), 5 ng/mL IL-7, and IL-15 (Prime Gene, Shanghai, China), as well as anti-CD3 and CD28 dynabeads (Gibco) with the ratio of 1: 1 for activating T cells. For bioimaging studies, T cells were transduced with a lentivirus expressing GFP and the click beetle green luciferase (CBG) 24 h after activation. Cells were counted and fed daily until day 10, when they were either used for functional assays or cryopreserved.

### Cytotoxicity assays

To assess CD19BiTE-mediated cytotoxicity, CFSE-labeled target cells (Raji, K562/CD19, K562) were cultured with 5 × 10^5^ T cells (E:T = 5) in 12-well plates. Mock, OVV, or OVV-CD19BiTE virus (10 MOI) supernatants (100 μl) or blinatumomab (100 ng/ml) were added. After 48 h of incubation, co-cultures we collected and stained with 7AAD (Thermo Fisher Scientific). Cells were analyzed by flow cytometry and the percentage of CFSE^+^/7AAD^+^ was determined. For bystander killing assays, Raji cells were infected with OVV-CD19BiTE or OVV at a MOI of 10 for 24 h. CD19-negative cells (K562) were cultured in the presence of T cells and its derivative K562/CD19 cells (E:T = 3) and OVV- or OVV-CD19BiTE-infected Raji cells were mixed with CFSE-labeled target cells (1:1). After 48 h, the cytotoxicity of the K562/CD19 and K562 were determined by flow cytometry. The percentage of CFSE^+^/7AAD^+^ cells was determined.

### Xenograft mouse models

All animal experiments were approved by the Ethics Committee for Animal Experimentation from Zhejiang University. Raji or Raji-luc were subcutaneously or intravenously injected into female, 8-week-old, NOD/scid/IL2rg −/− (NSG) mice (bred in house). To evaluate T-cell trafficking to the tumor, mice bearing Raji subcutaneous tumor were treated intratumorally with PBS, OVV, or OVV-CD19BiTE (2 × 10^7^ pfu/tumor). Twenty-four hours later, 1 × 10^7^ preactivated CBG99-luciferase-expressing T cells were intravenously injected into treated mice. In another parallel experiment, CBR-luciferase-GFP expressing Raji cells (1 × 10^5^ cells) were injected intravenously into NSG mice. Then PBS, OVV, OVV-CD19BiTE (2 × 10^7^ pfu), or blinatumomab (0.25 mg/kg) were injected intraperitoneally with a single dose, and CBG99-luciferase-BFP expressing human T cells (1 × 10^7^ cells) were injected intravenously. Mice were given an intraperitoneal injection of 15 mg/mL D-luciferin potassium salt solution (PerkinElmer) and imaged for indicated days using IVIS Lumina XRMS Imaging System (PerkinElmer).

For in vivo antitumor efficacy studies, mice bearing Raji subcutaneous tumor were treated intratumorally with PBS or the indicated viruses (2 × 10^7^ pfu/tumor) or blinatumomab (0.25 mg/kg) four times. Digital calipers were used to measure subcutaneous tumor diameter, with tumor volume being defined as: volume = length × width^2^ × 0.5. The body weight and tumor sizes of all mice were regularly monitored, and mice were killed by carbon dioxide suffocation if they exhibited acute weight loss or tumors ≥3000 mm^3^ in size. When tumors were no longer palpable, mice were considered to have achieved a CR. In another parallel experiment, CBG99-luciferase-expressing Raji cells were intravenously injected into NSG mice to establish disseminated xenograft mouse models of B-cell lymphoma. Then PBS, OVV, OVV-CD19BiTE (2 × 10^7^ pfu) or blinatumomab (0.25 mg/kg) were injected intraperitoneally three times. Every second day after OVV, OVV-CD19BiTE, or blinatumomab treatment, human T cells (1 × 10^7^ cells) were intravenously injected. Mice were given an intraperitoneal injection of 15 mg/mL D-luciferin potassium salt solution (PerkinElmer) and imaged for indicated days using IVIS Lumina XRMS Imaging System (PerkinElmer).

### Flow cytometry analysis of T-cell differentiation

Raji lymphoma cells were implanted intradermally into the right flanks of NSG mice (5 × 10^6^ cells). Mice bearing Raji subcutaneous tumor were treated intratumorally with PBS, OVV, OVV-CD19BiTE (2 × 10^7^ pfu/tumor), or blinatumomab (0.25 mg/kg). Twenty-four hours later, 1 × 10^7^ preactivated T cells were intravenously injected into treated mice. Tumors were harvested 3 days after the injection with forceps and surgical scissors and were weighed. They were then minced before incubation with Liberase (1.67 Wünsch U/mL) and DNase (0.2 mg/mL) in serum-free RPMI for 30 min at 37 °C. Cell suspensions were generated by mashing through a 70 μm nylon filter and then washed with complete RPMI. For tumor-infiltrating T-cells analysis, single-cell suspensions were generated and processed for surface labeling with anti-CD3, anti-CD45, anti-CD4, anti-CD8, anti-HLA-DR, anti-CD45RA, anti-CCR7, anti-CXCR3, and anti-CCR6 antibodies (Beckman). Live cells were distinguished from dead cells by using fixable dye eFluor506 (eBioscience). All flow data were acquired using either an LSRII flow cytometer (BD Biosciences). Data were analyzed with FlowJo software (Treestar).

### Statistical analysis

SPSS 17.0 (IBM Corp., NY, U.S.A.) and GraphPad Prism (GraphPad Software, Inc.) were used for statistical testing. Continuous data are given as means ± standard deviation (SD), and were compared through unpaired two-tailed Student’s *t*-tests or two-way analyses of variance (ANOVAs) with Tukey’s multiple comparisons test. Kaplan–Meier curves were used to assess survival outcomes. *P* < 0.05 was the significance threshold.

## Results

### Oncolytic vaccinia virus for expression of a BiTE targeting the CD19

**Fig. 1:**
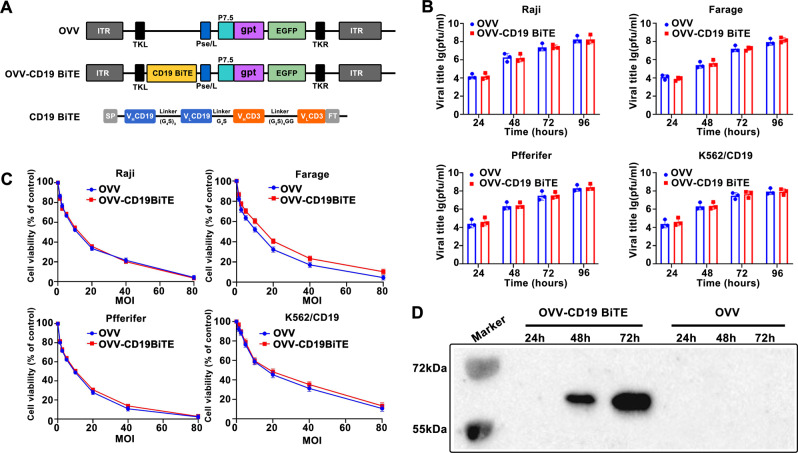
Generation of a CD19BiTE-expressing oncolytic vaccinia virus and its oncolytic properties in vitro. **A** Schematic structure representation of OVV-CD19BiTE. VL and VH domains of anti-CD19 and CD3Ɛ are connected by glycine and serine flexible linkers, flanked by the light chain immunoglobulin signal peptide (SP) and the FLAG tag (FT). CD19BiTE is inserted into the vaccinia virus TK gene under the control of the promoter Pse/l. TKR right flank sequences of thymidine kinase gene, TKL left flank sequences of thymidine kinase gene, gpt guanine phosphoribosyl transferase, EGFP enhanced green fluorescent protein, P7.5k vaccinia virus P7.5k early/late promoter, Pse/l synthesized vaccinia virus early/later promoter. **B** Viral production of OVV-CD19BiTE. Tumor cell lines (Raji, Pffeifer, Farage, K562/CD19) was infected with OVV or OVV-CD19BiTE at MOI of 0.5. At indicated time points, cell extracts were harvested and titrated by an TCID50-based method. Data represent the mean ± standard deviation (SD) of three independent experiments. **C** Comparative cytotoxicity profile of OVV-CD19BiTE. Raji, Pffeifer, Farage, and K562/CD19 cells were infected with serial dilutions of OVV or OVV-CD19BiTE. Cell viability was measured at 72 h post-infection. Data represent the mean ± standard deviation (SD) of three independent experiments. **D** The expression and secretion of CD19BiTE was detected by western blot assay. Cells were infected with OVV or OVV-CD19BiTE at an MOI of 2. After 24, 48, and 72 h post-infection, cell supernatants were collected and CD19BiTE was detected by an anti-FLAG antibody.

We have recently reported the generation of an OVV armed with therapeutic genes such as miR-34a, Smac, and Beclin-1, which showed enhanced antitumor efficacy in hematologic malignancy [12, 13]. In the present study, we engineered a novel OVV to express a CD19-targeting BiTE under the control of the vaccinia virus major later promoter Pse/l (denoted as OVV-CD19BiTE). The CD19BiTE was constructed by joining two scFv specific for human CD3ɛ and human CD19 with a flexible linker (GS linker) (Fig. 1A). To evaluate whether CD19BiTE insertion affects the replication of the virus, B-lymphoma cell lines Raji, Pffeifer, and Farage cell line, and K562/CD19 cell line was treated with an OVV that express GFP and OVV-CD19BiTE, respectively, and the replication kinetics of the viruses were determined by TCD50 mediated plaque-forming assays. There was no difference in the viral production of OVV versus OVV-CD19BiTE (*P* > 0.05) (Fig. 1B). We next assessed the killing kinetics of the viruses in dose-response cytotoxicity assays. As shown in Fig. 1C, the oncolytic potential was not impaired by the insertion of BiTE transgenes. In summary, compared with control OVV, the replication and oncolytic ability of OVV-CD19BiTE were unimpaired by the transgene.

To evaluate the secretion of the CD19BiTE by lymphoma cells upon viral infection and replication, cell-free supernatants of OVV-CD19BiTE-infected cells were analyzed SDS-PAGE and immunoblotting, which demonstrated BiTE expression and secretion (Fig. 1D). Furthermore, the secretion of the CD19BiTE was quantified from the supernatants of OVV-CD19BiTE infected cells by ELISA assay (Fig. S1). These results showed that the CD19BiTE was efficiently produced and released from OVV-CD19BiTE-infected cells.

### Supernatants from OVV-CD19BiTE-infected cells induce activation and proliferation of human T cells

**Fig. 2:**
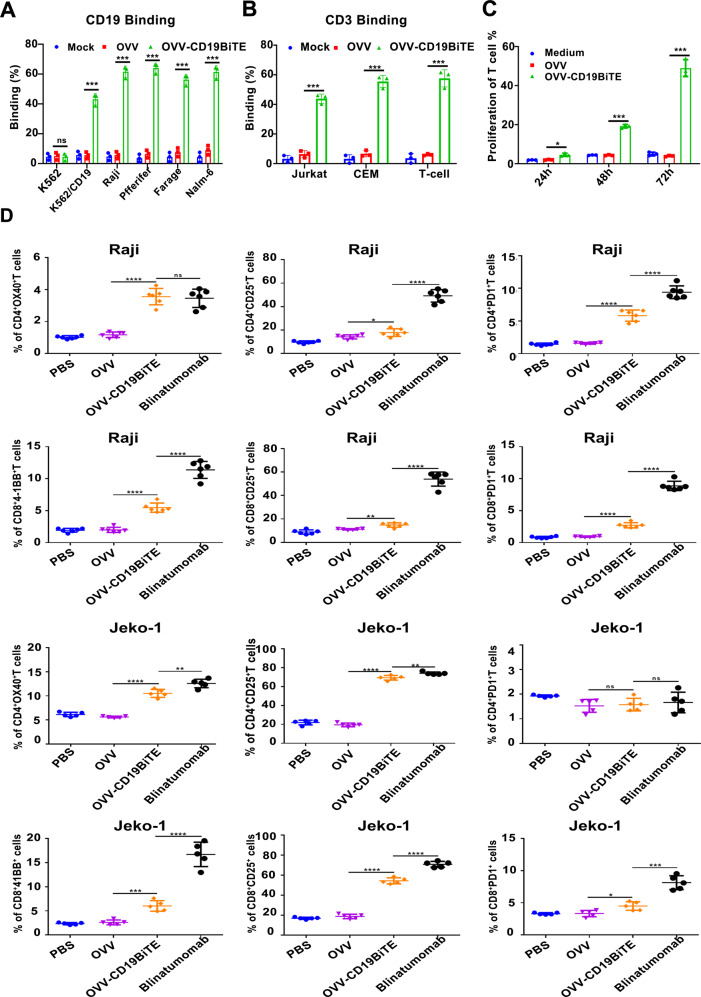
CD19BiTE released by OVV-CD19BiTE specifically binds to both target and effector cells and is able to activate T cells. CD19-positive cell lines including K562/CD19, Raji, Pffeifer, Farage, and Nalm-6 were co-cultured with supernatants of different viruses and K562 cells were used as control, while CD3-positive cell lines Jurkat, CEM, and human T cells were also incubated with mock, supernatants come from OVV, or OVV-CD19BiTE (100 μl). **A** CD19 binding and **B** CD3 binding were detected by flow cytometry using an anti-FLAG antibody. **C** The CFSE content (i.e., T-cell proliferation) on T cells was evaluated by flow cytometry following co-cultures with Raji cells (E:T = 5) and indicated supernatants (100 μl). **D** Human T cells were co-cultured with Raji or Jeko-1 cells in the presence of the different supernatants or blinatumomab (50 ng/ml). 48 h later, the expression levels of OX40, 4-1BB, CD25, and PD-1 on CD4^+^ and CD8^+^ T cells were assessed by flow cytometry. A representative result of triplicates is shown. *P < 0.05; **P < 0.01; ***P < 0.001, ****P < 0.0001; ns no significance, using one-way ANOVA test with post-hoc analysis.

**Fig. 3:**
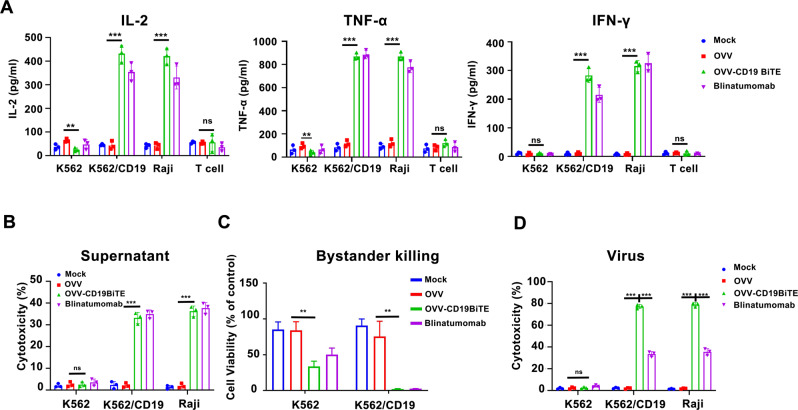
OVV-CD19BiTE-mediated oncolysis enhances T-cell function and induces a T-cell-mediated bystander effect. **A** Human T cells were co-cultured with or without K562, K562/CD19, or Raji cells, respectively, in the presence of the OVV supernatants (100 μl) or blinatumomab (100 ng/ml) for 24 h. Then, average concentration values of IFN-ɣ, TNF-α, and IL-2 cytokines in the supernatants obtained from this co-culture system were measured by ELISA assay. **B** Preactivated T cells were co-cultured with the indicated target cell lines (E:T = 5) in the presence of the OVV supernatants (100 μl) or blinatumomab (100 ng/ml). The percentage of cytotoxicity of target cells (CFSE^+^/7AAD^+^) was assessed by flow cytometry after 48 h co-culture. **C** CD19BiTE-mediated bystander tumor cell killing. CFSE-stained K562 cells or K562/CD19 were culture in the presence of T cells (E:T = 5), then the indicated supernatants (mock, OVV, OVV-CD19BiTE) and blinatumomab were added to this co-culture system. After 48 h, cytotoxicity of K562 cells and K562/CD19 cells were evaluated by flow cytometry. **D** Preactivated T cells were co-cultured with the indicated target cell lines (E:T = 5) in the presence of the OVV-CD19BiTE or OVV (MOI = 10) or blinatumomab (100 ng/ml). The percentage of cytotoxicity of target cells (CFSE^+^/7AAD^+^) was assessed by flow cytometry after 48 h co-culture. A representative result of triplicates is shown. ***P < 0.001; ns no significance, using one-way ANOVA test with post-hoc analysis.

Next, the functionality of the secreted BiTEs by the OVV was assessed by flow cytometry-based binding assays using a fluorescent-labeled anti-FLAG antibody. For this study, a panel of lymphoma cell lines, K562/CD19 cell line, K562, and CD3^+^ Jurkat, CEM, and human T cells were used. CD19 antigen-binding specificity was demonstrated only in the supernatants of OVV-CD19BiTE-infected cells and they specifically bound to CD19^+^ lymphoma cells and CD19^+^K562 cells, but not CD19^-^ K562 cells (Fig. 2A). The BiTEs also well bound to CD3^+^ Jurkat, CEM, and human T cells (Fig. 2B). To evaluate CD19BiTE-mediated T-cell effector functions, we investigated the ability of secreted BiTEs to activate PBMC-derived T cells by adding unstimulated human CD3^+^ cells to a culture of Raji cells. Supernatants from OVV-CD19BiTE-infected cells led to a significant increase in T-cell proliferation in a time-dependent manner, whereas the control OVV had no effect (Fig. 2C). Treatment with OVV-19BiTE supernatant or blinatumomab upregulated activation markers CD25, OX40, and 4-1BB on both CD4 and CD8 T cells and was also significantly induced PD-1 expression that is associated with T-cell exhaustion. However, blinatumomab had a stronger stimulation effect than secreted BiTE (Fig. 2D). Since the increase of cytokines is the response of T-cell activation and cytotoxicity, levels of IL-2, IFNγ, and TNFα were detected in the co-culture system. As shown in Fig. 3A, the concentrations of IL-2, IFN-γ, and TNF-α were 423.4 ± 30.21 vs 417.2 ± 28.17 pg/mL, 312.32 ± 33.43 vs 289.7 ± 22.3 pg/mL, and 878.21 ± 43.2 vs 879.43 ± 34.2 pg/mL in the supernatant obtained from Raji and K562/CD19 group, respectively, all of which were significantly higher than that of the control groups. Similar results were also observed in the co-culture in the presence of blinatumomab.

### Characterization and superior cytotoxicity of OVV-CD19BiTE in vitro

We next assessed whether the secreted CD19BiTE would kill B-lymphoma cells and whether the cytotoxicity by this CD19BiTE is antigen-specific. Cytotoxicity of human T cells in the presence of supernatants from OVV-infected cells and OVV-CD19BiTE, or blinatumomab, respectively, were measured in three different cancer cell lines. The secreted CD19BiTE and blinatumomab elicited strong cytotoxicity in Raji and K562/CD19 cells, but not in K562 cells, indicating that the secreted CD19BiTE-mediated cytotoxicity was dependent on CD19 expression (Fig. 3B). Recent studies demonstrated that BiTEs could also mediate a bystander killing of adjacent tumor cells [17]. To determine whether CD19BiTE mediate the bystander effect, CFSE-stained K562 cells or K562/CD19 were co-cultured with human T cells. Then the supernatants obtained from OVV or OVV-CD19BiTE-infected cells or blinatumomab were added to this co-culture system. The cell death in CFSE-labeled target cells was assessed by flow cytometry. As shown in Fig. 3C, we observed obvious cytotoxicity of K562 cells only when co-cultured together with OVV-CD19BiTE supernatants or blinatumomab. This result supports an existing BiTE-dependent T-cell-induced bystander lysis of CD19-negative cells. To investigate the enhanced antilymphoma efficacy mediated by both oncolysis and the secreted CD19BiTEs, human T cells were co-culture with Raji, K562^CD19-positive^, and K562 cells, which were stained by CFSE, in the presence of blinatumomab, OVV-CD19BiTE at an MOI of 2 or control OVV (MOI = 2), respectively. In Raji and K562/CD19 cells, OVV-CD19BiTE treatment elicited significantly increased cell death (*p* < 0.001 for Raji and K562/CD19) compared with blinatumomab treatment (Fig. 3D), demonstrated superior in vitro CD19^+^ tumor cytotoxicity.

### OVV-CD19BiTE are capable of recruiting an immune response to a tumor and stimulating T-cells proliferation

**Fig. 4:**
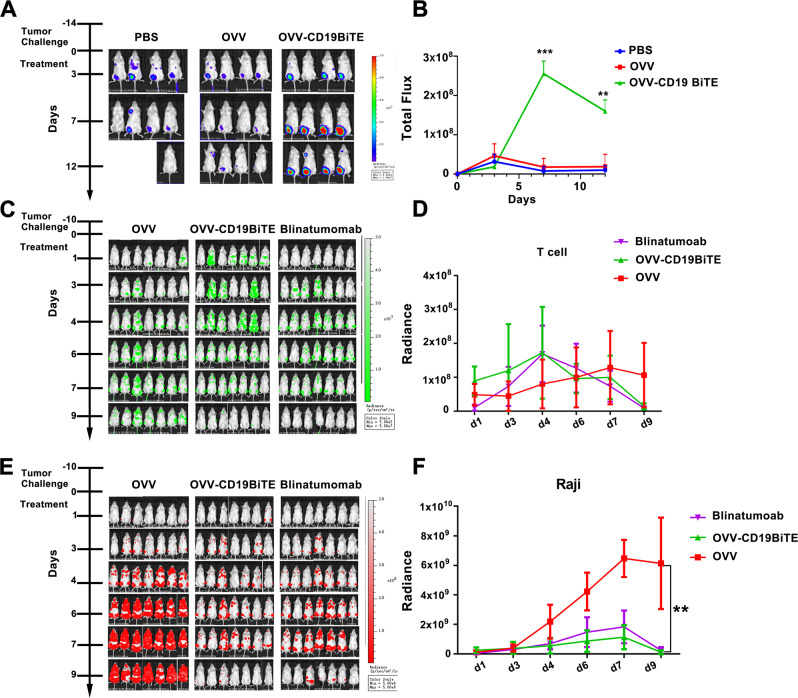
OVV-CD19BiTE induces accumulation and increased persistence of tumor-infiltrating T cells in vivo. **A** NSG mice bearing subcutaneous Raji tumors were intratumorally injected with PBS, OVV, or OVV-CD19BiTE (2 × 10^7^ pfu/tumor). 24 h later, mice received an intravenous injection of preactivated T cells expressing luciferase gene (T-luc cells, 1 × 10^7^cells/mouse). Images of respective mice were shown. **B** Luciferase activity was analyzed by bioluminescence imaging (IVIS) until day 12. Mean values ± SD with 4 animals per group are shown. **P < 0.01; ***P < 0.001, OVV-CD19BiTE significant by one-way ANOVA test with post-hoc analysis compared to PBS and OVV groups. **C**–**F** CBR-luciferase-GFP expressing Raji cells were injected intravenously into NSG mice on Day −10, then injected i.p. with a single dose of PBS, OVV, OVV-CD19BiTE (2 × 10^7^ pfu) or blinatumomab (0.25 mg/kg) and injected intravenously with CBG99-luciferase-BFP expressing human T cells on Day 0. Images of respective mice about T-cell stimulation (**C**) and tumor progression (**E**) were shown. Luciferase activity of T cells (**D**) and Raji cells (**F**) was analyzed by bioluminescence imaging. A representative result of triplicates is shown. **P < 0.01, using one-way ANOVA test with post-hoc analysis.

It is known that BiTEs are uniquely designed both to redirect and recruit T cells to tumor tissues [23–25]. In this study, T lymphocytes that infiltrated into tumor sites were investigated. For this purpose, Raji cells were engrafted in the subcutaneous flank of NSG mice and then PBS, OVV, or OVV-CD19BiTE were intratumorally injected, respectively. Twenty-four hours later, luciferase-expressing human T cells were given by intravenous injection (IV). Bioluminescence (BLI) images showed that there was straightforward recruitment of T lymphocytes in tumors of OVV-CD19BiTE treated mice but not of the control groups (Fig. 4A, B), suggesting that infused T cells were effectively recruited to the tumor sites.

To investigate T-cell proliferation and its relationship with antitumor activity in vivo, mice bearing carcinomatosis established by vein injection of Raji cells were treated with a single dose of OVV, OVV-CD19BiTE, or blinatumomab, respectively, and then human T cells that expressed CBG99-luciferase-BFP were also infused into the treated mice. The peak levels of T-cell expansion were higher in both OVV-CD19BiTE and blinatumomab group compared with OVV, which was observed in 4 days (Fig. 4C, D). Meanwhile, the treatment with either OVV-CD19BiTE or blinatumomab was able to significantly inhibit tumors developed by Raji cells (Fig. 4E,F).

### OVV-CD19BiTE confers superior antilymphoma activity compared with blinatumomab in vivo

**Fig. 5:**
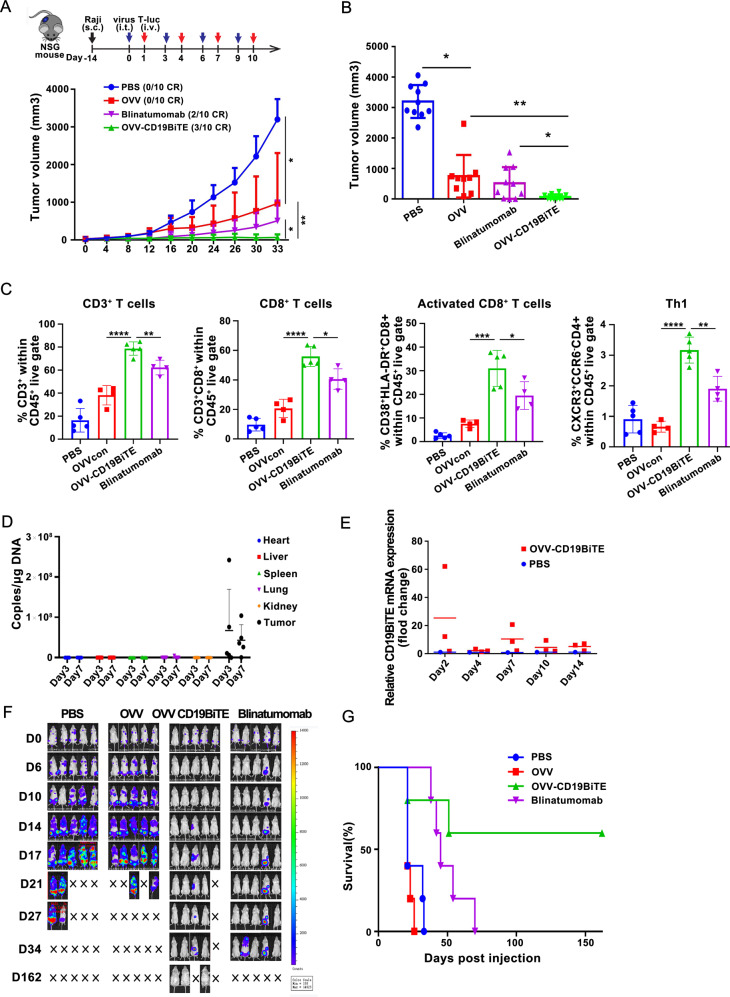
OVV-CD19BiTE shows improved antitumor efficacy in vivo. **A** NSG mice bearing subcutaneous Raji tumors were treated with PBS, OVV, OVV-CD19BiTE (1 × 10^7^ pfu/tumor, I.T.) or blinatumomab (0.25 mg/kg, I.P.) every 3 days for four times. 24 h after virus treatment, animals were infused with preactivated T cells (1 × 10^7^ cells, I.V.) for four times. The mean tumor growth ±SD is shown (*n* = 10). **B** At the end of the experiment about panel **A** (Day 33), tumor volumes were compared among groups. **C** In another parallel experiment, NSG mice bearing subcutaneous Raji tumors were treated with PBS, OVV, OVV-CD19BiTE (2 × 10^7^ pfu/tumor, I.T.) or blinatumomab (0.25 mg/kg, I.P.). On day 3 after virus injection, tumors were harvested and cell suspension was prepared. Percentage of CD3, CD8, CD4, naïve CD8, and Th1 cells were analyzed by flow cytometry. *P < 0.05; **P < 0.01; ***P < 0.001; ****P < 0.0001; ns no significance, using one-way ANOVA test with post-hoc analysis. **D** On day 3 and 7 after virus injection, the DNA content of CD19BiTE in heart, liver, spleen, lung, kidney, and tumor was detected by qPCR. **E** The mRNA level of CD19BiTE of tumors was detected by qRT-PCR. **F** NSG mice model of systemic tumorigenesis of Raji-luc cells were intraperitoneally injected with PBS, OVV, OVV-CD19BiTE (1 × 10^7^ pfu/tumor) or blinatumomab (0.25 mg/kg, I.P.) every three days for four times. 24 h after every virus treatment, animals were treated with preactivated T cells (1 × 10^7^ cells, I.V.) for four times. Luciferase activity of Raji-luc was analyzed by bioluminescence imaging (IVIS) at indicated day until day 162 (*n* = 5). **G** Kaplan–Meier analysis was performed to assess the statistical difference between different treatment groups. **P < 0.01, using one-way ANOVA test with post-hoc analysis.

After demonstrating the efficacy of OVV-CD19BiTE in vitro, we moved forward to determine the antilymphoma activity of this novel OVV product in vivo using two distinct models in NSG mice. In the first model, Raji cells were injected subcutaneously leading to xenografts. The mice bearing established tumors (tumor volume reached 80–100 mm^3^) received intratumoral (IT) injection of OVV, OVV-CD19BiTE, or intraperitoneal (IP) injection of blinatumomab, respectively. PBS was used as a negative control. One day later, human T cells were IV administered to treated mice. As shown in Fig. 5A, OVV-CD19BiTE-treated tumors were substantially delayed in their growth. At 33 days after initiation of the treatment, all mice were euthanized and tumors were excised, and then tumor volumes were calculated. Xenografts of mice treated with OVV-CD19BiTE only have an average tumor volume of 64.3 mm^3^. In contrast, mean tumor volumes were 3200.3, 967.2, and 509.1 mm^3^ in PBS-, OVV-, and blinatumomab-treated groups, respectively (Fig. 5B). Importantly, complete elimination of tumors was observed in 3/10 mice treated with OVV-CD19BiTE, and 2/10 mice treated with blinatumomab, respectively.

To understand why OVV-CD19BiTE is much more effective than OVV or blinatumomab in generating antitumor effects, we performed another parallel animal experiment and investigated the difference in number and phenotype of T cells within tumors across the treatment groups. After establishing Raji-xenograft, mice were randomized into treatment groups that received intratumoral administration of PBS, OVV, OVV-CD19BiTE, or intraperitoneal administration of blinatumomab, respectively, and then human PBMC were also administrated by intravenous infusion. Three days later, the tumors were harvested to obtain cell suspensions. Tumors treated with OVV-CD19BiTE and blinatumomab showed an increased percentage of infiltrating T cells that included various phenotypes such as CD4, CD8, naïve CD8 (CD8^+^ CD45RA^+^ CCR7^+^), central memory CD8 (CD8^+^ CD45RA^−^ CCR7^+^), effector memory CD8 (CD8^+^ CD45RA^−^ CCR7^−^), effector CD8 (CD8^+^ CD45RA^+^ CCR7^+^), Th1 (CD4^+^ CXCR3^+^ CCR6^−^), and Th2 (CD4^+^ CXCR3^−^ CCR6^−^) cells when compared with OVV or PBS. Importantly, OVV-CD19BiTE contributed to a more significantly increased percentage of CD3, CD8, and naïve CD8 T cells in contrast to blinatumomab (Fig. 5C and Fig. S2).

To evaluate the speciality of the biodistribution of OVV-CD19BiTE and the expression of CD19BiTE after intratumoral injection, an independent animal study was conducted. Ten mice bearing xenografts were treated with a single dose of OVV-CD19BiTE. At different time points after injection, the treated mice were selected randomly to be killed, and expressions of the virus gene and mRNA of CD19BiTE in tumor tissues were determined by PCR assays. The expression of the virus gene was detected in tumor tissues but not in normal tissues including the heart, liver, spleen, lung, and kidney (Fig. 5D). The mRNA expression of CD19BiTE could be detected in tumors up to 14 days post-treatment with a single dose of OVV-CD19BiTE (Fig. 5E).

In another model, IV carcinomatosis was modeled in NSG mice via intravenous injection with luciferase-expressing Raji cells. Mice were treated 7 days later with I.P. injection of PBS, OVV, OVV-CD19BiTE, or blinatumomab as indicated. OVV-CD19BiTE and blinatumomab, but not OVV, led to marked tumor regression as demonstrated by serial BLI imaging (Fig. 5F). Moreover, OVV-CD19BiTE was most effective for controlling Raji tumor cell growth, and three of five mice treated with OVV-CD19BiTE remained tumor-free for up to 162 days post-treatment, which resulted in significantly improved overall survival compared to OVV or even blinatumomab treatment (Fig. 5F, G).

## Discussion

Current developments in immunotherapy using BiTEs show remarkable efficacy in certain subgroups of cancer patients. Blinatumomab is an anti-CD19BiTE with excellent cell-binding capacities. It was approved for use in patients with R/R B-cell precursor ALL [25]. In a multicenter phase 3 trial, R/R B-cell precursor ALL patients were randomized to receive blinatumomab or chemotherapy. The results demonstrated blinatumomab was superior to chemotherapy in overall survival. However, AEs of grade 3 or higher were found in 87% of the patients treated with blinatumomab [5]. Despite the success of anti-CD19/CD3 BiTE, many biological obstacles, such as toxicity, short half-life in the serum, and immunosuppressive TME, have limited the use of anti-CD19/CD3 BiTE therapy in the clinic. Therefore, novel strategies are required to improve not only its efficacy but also its safety.

Oncolytic viruses are ideal vehicles for tumor-specific delivery of BiTEs as immunotherapeutic transgenes because encoding BiTEs in OVs can overcome BiTE limitation and this combination promises mutual benefits [23, 24]. Recently, OV vectors from four kinds of virus families including adenovirus, measles virus, herpes simplex virus-1, and vaccinia virus were used in OV-BiTE studies [16–19, 26, 27]. In the present study, we have developed a strategy to address some limitations of anti-CD19/CD3 BiTE by establishing a novel OV-BiTE using an oncolytic vaccinia virus that encodes within it a CD19-specific BiTE for secretion by infected B-cell lymphoma cells. Our data demonstrate the therapeutic efficacy of OVV-CD19BiTE against B-cell lymphoma, and show that insertion of BiTE expression cassettes into OVV did not compromise replicative and oncolytic capacities. To our knowledge, this is the first study to evaluate an anti-CD19/CD3 BiTE-armed OVV in preclinical lymphoma models.

OVV-CD19BiTE are functional in terms of antigen-binding specificity, target-specific T-cell activation and proliferation, and induction of T-cell cytotoxicity. It exerted superior in vitro and in vivo CD19^+^ tumor cytotoxicity compared to control OVV or blinatumomab. Mechanistically, OVV-CD19BiTE is capable of recruiting T cells to a tumor and stimulating its proliferation, even in the absence of exogenous IL-2, compared with its parental OVV. Both viral and CD19BiTE mRNA were detected in tumor tissue at day 3 and day 7, respectively, post a single dose of i.t. virus injection. In a previous study of oncolytic measles viruses encoding anti-CEA/CD3 BiTE (MV-BiTE) that was used to treat colorectal tumor-bearing NSG mice on four consecutive days, Speck et al [26] reported that intratumoral viral gene and BiTE expression were demonstrated until 10-day post-treatment. Collectively, these data indicate that OV-BiTE can induce robust tumor-specific viral propagation and secretion of BiTE. Our study demonstrates that the therapeutic efficacy of BiTEs against B-cell lymphoma can be achieved by means of an oncolytic viral vector (OVV-CD19BiTE) and shows long-term tumor remissions without relapse after OVV-CD19BiTE treatment, which may attribute to immunomodulatory effects of oncolysis in combination with local BiTE-mediated T-cell recruitment and T-cell activation [26, 27]. OV infection could cause IFN response followed by the release of T-cell-recruiting cytokines and chemokines in the TME, thereby increasing infiltration of the T cells in a variety of ways [11]. OV-BiTE also had been demonstrated to increase T-cell infiltration and expansion [17]. In this study, significantly increased infiltration and expansion of T cells were found in subcutaneous tumors treated with the OVV-CD19BiTE after intravenous T-cell infusion. Another noteworthy finding in our in vivo experiments was that OVV-CD19BiTE-treated tumors harbored high numbers of T cells, with 55.9% CD8^+^ tumor-infiltrating lymphocytes. Treatment with OVV-CD19BiTE also led to significantly increased naïve CD8 T subpopulation within tumors compared to OVV and blinatumomab treatment. This difference may contribute to the superior antitumor activity of OVV-CD19BiTE because it was demonstrated that naïve and memory T-cells surpass effector T cells in induction of potent antitumor responses for adoptive cell therapy [28]. However, to elucidate the interaction between the OVV-CD19BiTE and the immune cells within TME, further studies using an immunocompetent mouse model, but not NSG mouse model, are required.

Systemic delivery is a major goal in the field of virotherapy, which is the preferred route of administration in many clinical situations for the oncolytic virus to maximize the delivery of the virus to metastatic cancers [29]. Several preclinical and clinical trials have revealed the remission of disseminated cancer after systemic oncolytic virotherapy [30–33]. In this study, a mouse model with disseminated B-cell tumors was established by intravenous injection of Raji cells. Treatment with OVV-CD19BiTE delivered intraperitoneally significantly suppressed the growth of Raji tumors compared with control OVV and blinatumomab, and led to a substantial response, surprisingly, three of five mice (60%) given OVV-CD19BiTE achieved long-term tumor remissions without relapse and had very long survival.

In summary, our findings provide preclinical evidence for the therapeutic potential of OVV-CD19BiTE, and show that OVV-CD19BiTE activated human T cells, induced recruitment of T cells, and significantly increased naïve and central memory T subpopulations within TME, thereby enhancing antilymphoma activity in preclinical models. Thus, this novel OVV could circumvent limitations in current BiTE therapy and may translate into meaningful therapeutic effects in the treatment of B-cell lymphomas.

## Declarations

The animal experiments were approved by the Institutional Animal Care and Use Committee, Zhejiang University. The studies involving human participants were reviewed and approved by an independent Ethics Committee of The Second Affiliated Hospital, College of Medicine, Zhejiang University. The participants provided their written informed consent to participate in this study.

## Supplementary information

supplementary Figure Legends

Figure S1

Figure S2

## Data Availability

The datasets generated and/or analyzed during the current study are available from the corresponding author on reasonable request.
