# Supplementary material for: CD19-targeted BiTE expression by an oncolytic vaccinia virus significantly augments therapeutic efficacy against B-cell lymphoma
Source: Blood Cancer J. 2022 Feb 28;12(2):35. doi: 10.1038/s41408-022-00634-4 (PMC8885649; doi:10.1038/s41408-022-00634-4)
Supplement: Supplementary file 1 — supplementary Figure Legends [file 41408_2022_634_MOESM1_ESM.docx]

**Supplementary Figure legends**

**Supplemental Figure 1. Quantification of secreted CD19BiTE protein in vitro.** Human CD3 or CD19 proteins were coated on the ELISA plates respectively, CD19BiTE protein (purified from cell-free system) was tittered as standard sample and the curve was fitted (A) and (B); The protein level was quantified from the supernatants of OVV-CD19BiTE infected cells (0.1 MOI for Hela-S3 and 10 MOI for Raji, C and D).

**Supplemental Figure 2. T cell subsets within tumors.** Flow cytometric analysis of tumor-infiltrating T cells (TILs) collected from Raji subcutaneous tumor after treatment with PBS, OVV, OVV-CD19BiTE (2 × 10^7^ pfu/tumor) or Blinatumomab (0.25mg/kg) and 1 × 10^7^ preactivated T cells (I.T.). *, P < 0.05; **, P < 0.01; ***, P < 0.001; ****, P < 0.0001; ns, no significance, using one-way ANOVA test with post hoc analysis.
