# Supplementary figures and images for: CD19-targeted BiTE expression by an oncolytic vaccinia virus significantly augments therapeutic efficacy against B-cell lymphoma
Source: Blood Cancer J. 2022 Feb 28;12(2):35. doi: 10.1038/s41408-022-00634-4 (PMC8885649; doi:10.1038/s41408-022-00634-4)

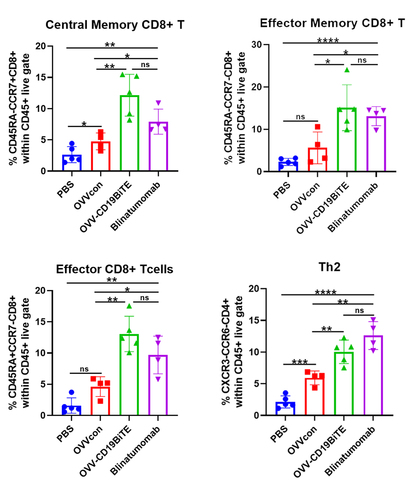

Supplement: Supplementary file 3 — Figure S2 [file 41408_2022_634_MOESM3_ESM.jpg]
